# Benign Epithelial Salivary Neoplasms: Single-Centered Histopathologic and Clinicodemographic Romanian Retrospective Study

**DOI:** 10.3390/clinpract15120235

**Published:** 2025-12-15

**Authors:** Constantin Aleodor Costin, Adriana Grigoraș, Elena Corina Andriescu, Cornelia Amalinei

**Affiliations:** 1 Department of Morphofunctional Sciences I, Grigore T. Popa University of Medicine and Pharmacy Iasi, 700115 Iasi, Romania; aleodor.costin@umfiasi.ro; 2 Department of Histopathology, Institute of Legal Medicine, 700455 Iasi, Romania; 3 Department of Pathology, “Sf. Spiridon” County Hospital, 7000111 Iasi, Romania; andriescu_corina@yahoo.co.uk

**Keywords:** benign epithelial salivary gland tumors, epidemiology, parotid gland, pleomorphic adenoma

## Abstract

**Background**: Epidemiological studies on benign epithelial salivary gland tumors are challenging due to their rarity, pathological heterogeneity, variable tumor locations, and the limited national data collection in Romania. Our study aimed at the evaluation of benign epithelial salivary gland tumors collected over fifteen years in a tertiary center, in order to characterize their demographic and histopathological profiles and to contribute to their diagnostic and therapeutic strategies. **Materials and Methods**: A retrospective analysis of 404 cases of benign epithelial salivary gland tumors diagnosed in “Sf. Spiridon” County Hospital, Iasi, from 2010 to 2024, has been performed. **Results**: The analyzed cases showed a slight female predominance (52.97%) and a mean patient age of 54.55 ± 14.207 years. Tumor frequency increased progressively with age, peaking in the sixth and seventh decades of life. The most common histological types were pleomorphic adenoma (62.62%) and Warthin tumor (29.95%), both types showing a predominant parotid gland involvement (88.51%). The recurrences were rare, being registered only in 1.58% of pleomorphic adenomas. A significant association between tumor histological type and both gender (*p* < 0.001) and age group (*p* < 0.001) was registered, while no significant correlation between gender and age group (*p* = 0.288) or between tumor location and gender or age group (*p* = 0.382; *p* = 0.383) was found. **Conclusions:** The frequency of pleomorphic adenoma is increasing, showing an age-related distribution and parotid gland propensity. Key morphological features in each histological type support a better preoperative stratification, a more confident margin assessment, and an individualized extent of excision with function preservation.

## 1. Introduction

Salivary gland neoplasms represent a heterogeneous group of tumors with a relatively low incidence, accounting for approximately 3–6% of all head and neck tumors [1]. Globally, the estimated annual incidence ranges from 0.4 to 13.5 cases per 100,000 individuals [2,3]. They are characterized by histopathological variability and extensive anatomical distribution, involving both major and minor salivary glands located in the oral cavity and upper aerodigestive tract [1,3,4]. Although most of the tumors arise in the parotid gland, they are also commonly diagnosed in the submandibular glands or in the minor salivary glands [5,6]. Although benign lesions outnumber the malignant ones, most of the epidemiological studies have been focused on malignant salivary gland tumors [1].

Their relative low incidence, added to their wide morphological type and location heterogeneity, leads to significant methodological challenges in epidemiological studies [1]. Moreover, the wide histopathological spectrum of salivary gland tumors raises significant diagnostic difficulties, added to their diverse clinical manifestations [7].

In this context, our study provides an in-depth evaluation of benign epithelial salivary gland tumors in our geographic region, collected over fifteen years, being diagnosed and treated in a tertiary center, in order to characterize the patients’ demographics and the tumors’ histopathological features, contributing to diagnostic and therapeutic strategies.

## 2. Materials and Methods

**Figure 1:**
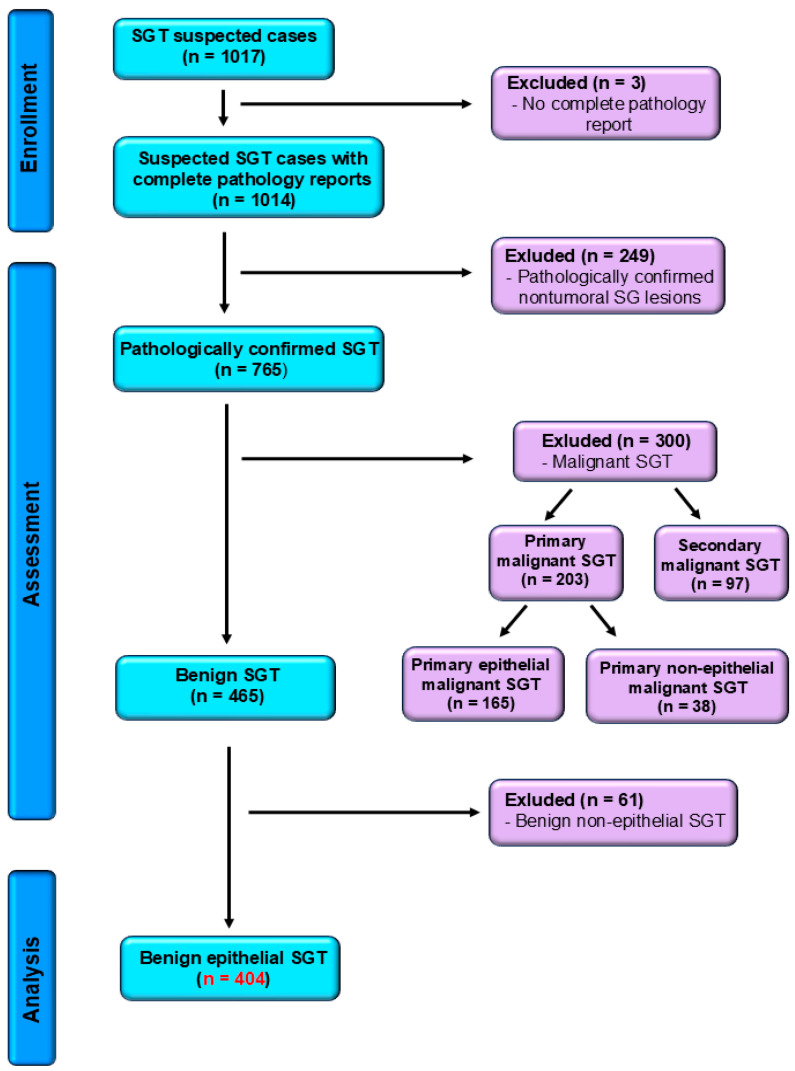
STROBE flow diagram for benign salivary gland tumors confirmed cases. SGT—salivary gland tumors.

Our retrospective study, conducted over a fifteen-year period, spanning from 1 January 2010 to 31 December 2024, was carried out in the Department of Pathology of “Sf. Spiridon” County Hospital, Iasi. A total of 569 cases of benign and malignant primary epithelial salivary gland tumors were registered, based on surgically excised specimens submitted for routine histopathological assessment. The eligibility criteria comprised the confirmed histopathological diagnosis of benign epithelial salivary gland tumors and microscopy findings, along with well-preserved formalin-fixed paraffin-embedded (FFPE) tissue blocks and accurate demographical information. The exclusion criteria were the following: uncertain diagnosis, inadequate archived tissue, malignant type of salivary gland tumors, non-epithelial benign salivary gland tumors, or the lack of complete patient information. Following the application of the exclusion criteria, 404 cases of benign epithelial salivary gland tumors were eligible for analysis (Figure 1), with histopathological re-evaluation and reclassification according to the latest WHO Classification [8].

According to salivary gland tumors’ characteristics and location, the surgical management consisted in extracapsular dissection (ECD), superficial parotidectomy (SP), and total parotidectomy (TP) in parotid tumors; total sialoadenectomy (TS) in submandibular and sublingual glands tumors; and wide local excision (WLE) in minor salivary glands tumors, without any enucleation performed in the study group.

Intraoperative salivary imprint cytology was used in selected cases, with samples of freshly cut tumor surfaces and/or surgical tumor margins, either for rapid triage in suspected malignancy or for resection accuracy assessment. The protocol consisted of two to four May–Grünwald–Giemsa (MGG) stained imprints per tumor, associated with intraoperative diagnosis, while their findings were correlated with FFPE hematoxylin–eosin (H&E) section features.

Immunohistochemical (IHC) analysis performed on FFPE tissue sections used a standard streptavidin–biotin–peroxidase technique with endogenous biotin blocking and 3,3′-Diaminobenzidine (DAB) as chromogen, with the following primary antibodies: AE1/AE3, CK7, CK5/6, and CEA as epithelial markers; p63, SMA, calponin, S100, CD10, and GFAP as myoepithelial markers; and androgen receptor (AR), estrogen receptor (ER), HER2, CD117, synaptophysin, chromogranin, and Ki-67 as relevant markers for the tumors characterization, with appropriate positive and negative controls included in each protocol.

The data analysis was performed using SPSS version 25 (IBM, Armonk, NY, USA) and Microsoft Excel 2016. Descriptive statistics (counts, percentages, mean, and standard deviations) were calculated. The normality of quantitative variables was assessed by the Kolmogorov–Smirnov test, with the null hypothesis not rejected at *p* > 0.05, showing a normal distribution. Correlations between demographic and histopathological variables were assessed by the Pearson chi-square test. *p*-value < 0.05 was established as statistically significant for all tests. The strength of significant associations was assessed by Cramer’s V (V), using standard interpretation criteria (weak = 0.10, moderate = 0.30, and strong > 0.50). The ethical approval was obtained both from the Ethics Committee of Grigore T. Popa University of Medicine and Pharmacy Iasi (approval no. 500) on 30 November 2024 and from the Ethics Committee of “Sf. Spiridon” County Hospital Iasi (approval no. 99) on 8 November 2024.

## 3. Results

### 3.1. Epidemiological Distribution

The predominant female gender was registered in the study group, with 214 cases (52.97%), compared to 190 male cases (47.03%), with a women-to-men ratio of 1.12:1.

**Figure 2:**
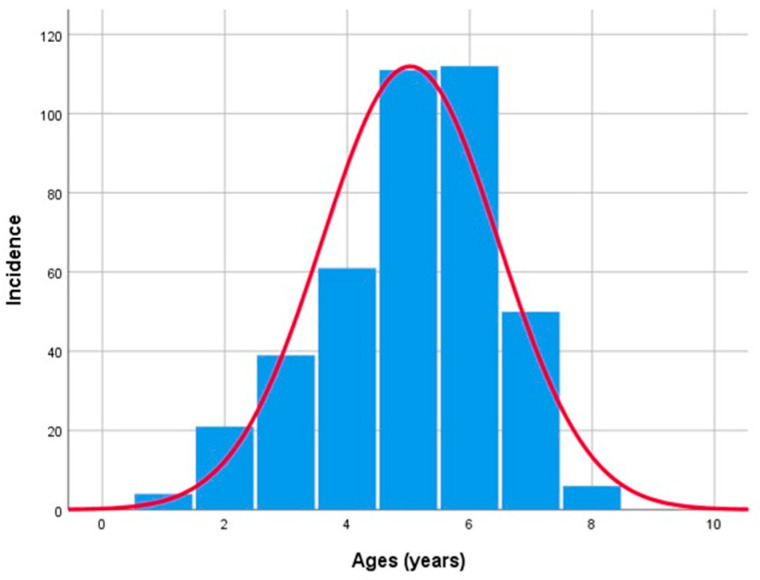
Benign epithelial salivary gland tumors distribution by age.

The mean patients’ age group was 54.55 ± 14.207 years, showing relatively similar gender ranges, with 52.8 ± 13.65 years in women and 56.52 ± 14.59 years in men. The oldest patient included in the study was 89 years old, while the youngest was 10 years old, and the highest number of cases was observed in the 60–69 age group (27.72%) (Figure 2). 

**Figure 3:**
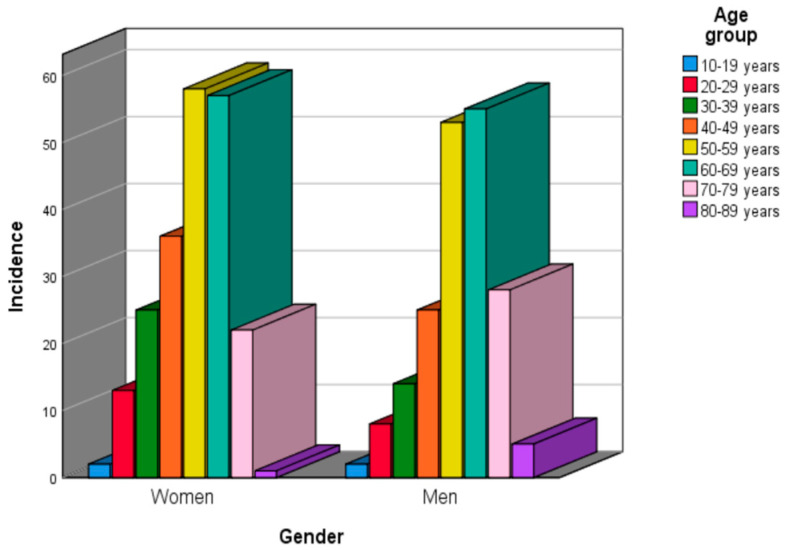
Benign epithelial salivary gland tumors distribution by gender and age group.

Regarding the age groups, according to gender distribution, 58 patients (14.35%) were in the 50–59 age group, followed by patients in the 60–69 age group (57 cases, representing 14.10% and 36 cases, representing 8.91%, respectively), in women. Most male patients were included in the 60–69 and 50–59 age groups (55 cases; 13.61% and 53 cases; 13.11%) (Figure 3). However, no statistically significant association was found between patients’ gender and their age groups (*p* = 0.288; effect size: V = 0.145).

### 3.2. Tumor Location

The analysis of the tumors’ location revealed a propensity for parotid gland location, together accounting for 88.85% of all cases included in our study. Tumors of the submandibular glands showed a significantly lower frequency, with about 4% of tumors in each submandibular gland. Lesions involving the palatine glands and other minor salivary glands within the oral cavity or sublingual glands were extremely rare (Table 1).

The analysis of tumor distribution by age group and location showed a higher number of cases of parotid tumors across all age groups. However, no statistically significant association was detected between benign epithelial tumor location and gender or patients’ age groups (*p* = 0.382 vs. *p* = 0.383; effect size: V = 0.126 vs. V = 0.135).

### 3.3. Tumor Histopathological Type Distribution

On gross examination, benign salivary gland tumors generally appeared as well circumscribed, encapsulated masses, with a firm to rubbery consistency, measuring 9–85 mm in diameter with a mean of 31.92 ± 12.99 mm.

The analysis of the benign epithelial salivary gland tumors’ histological type confirmed the predominance of pleomorphic adenoma, with 253 cases (62.62%), followed by Warthin tumor, with 121 cases (29.95%) (Table 2). Other benign tumors, such as myoepithelioma (13 cases, 3.21%), basal cell adenoma (9 cases, 2.22%), oncocytoma (5 cases, 1.23%), and canalicular adenoma (3 cases, 0.74%) were registered in a considerably reduced amount compared to the other tumor types.

The histological distribution of benign salivary gland tumors showed a marked predominance of pleomorphic adenoma among women compared to men (161 cases, 39.85% vs. 92 cases, 22.77%), while other histological types showed no significant gender-related propensity (Table 2). In contrast, Warthin tumors, with 121 cases (29.95%), displayed a male gender propensity, with 82 cases in men vs. 39 cases in women. Moreover, the statistical analysis revealed a significant association between gender and the benign epithelial tumor histological type in the studied cohort (*p* < 0.001; effect size: V = 0.294).

Furthermore, pleomorphic adenoma was the most common tumor across all age groups, with peak occurrence in patients aged between 40 and 69 years. 

Following pleomorphic adenoma, Warthin tumor was most frequently diagnosed in the seventh decade of life, with 46 (11.38%) of cases, while myoepithelioma and oncocytoma were rare, and most basal cell adenomas were identified in the seventh decade of life (6 cases, 1.48%) (Table 2). Only four cases were identified in patients under 20 years, being diagnosed as pleomorphic adenomas. 

Considering that most benign salivary gland tumors were diagnosed in the sixth and seventh decades of life, an increased occurrence according to age was noticed (Table 2). The statistical analysis revealed a significant strong association (*p* < 0.001) between the histopathological type and the patients’ age groups, with a large effect size (Cramér’s V = 0.527).

Moreover, pleomorphic adenoma was the most frequent histological type, involving the right parotid gland in 114 patients (28.21%) and the left parotid gland in 104 patients (25.74%). Warthin tumor represented the second most common diagnosis, showing a similar distribution to pleomorphic adenoma, involving about 14% in each parotid gland.

Myoepitheliomas were also predominantly located in the parotid glands, with isolated cases in the palatine glands (two cases, 0.51%) and in the right submandibular and sublingual glands, with one case each (0.24%). Oncocytomas and basal cell and canalicular adenomas were exclusively located in the parotid glands (Table 2).

Tumors arising in the minor salivary glands were rare, with only 11 registered cases (2.72%), 8 of them being diagnosed as palatine gland pleomorphic adenomas.

Sublingual gland tumors were almost absent, with only a myoepithelioma identified in the right sublingual gland, while the submandibular glands harbored 33 tumors (8.16%), the majority being pleomorphic adenomas. The statistical analysis demonstrated a significant association between the histological types of benign salivary gland tumors and tumor location (*p* = 0.004; effect size V= 0.165).

### 3.4. Tumor Histopathological, Cytological, and Immunohistochemical Findings 

**Figure 4:**
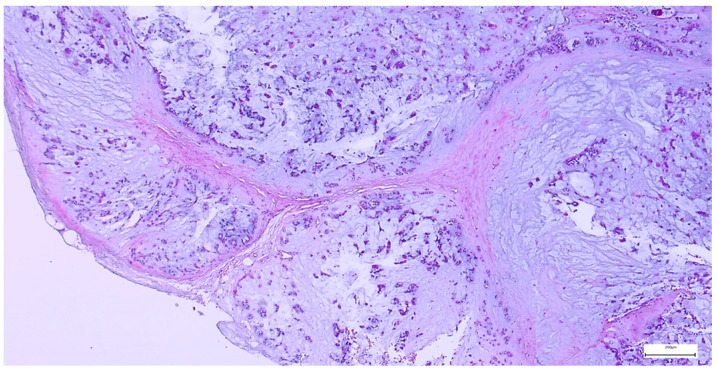
Multinodular, biphasic tumor cells proliferation, embedded in a chondromyxoid stroma and surrounded by a thin hypocellular fibrous capsule in pleomorphic adenoma (H&E staining, 5×).

On gross examination, most pleomorphic adenomas appeared as well-demarcated, bosselated, gray-white myxoid masses. Intraoperative salivary imprint cytology was applied if tumors contained areas of necrosis, hemorrhages, or infiltrative margins. Tumor sizes ranged from 9 to 82 mm, with a mean diameter of 32.55 ± 13.38 mm. Histological examination of pleomorphic adenomas revealed the characteristic biphasic epithelial–myoepithelial architecture. The tumors displayed a heterogeneous cellular composition (248 cases; 98.02%), while the pleomorphic adenoma oncocytic subtype was identified in five cases (1.98%) (Table 3). The stromal component exhibited marked variability, the myxoid phenotype being the predominant pattern (138 cases; 54.54%), followed by chondromyxoid phenotype (72 cases; 28.45%) (Figure 4 and Table 3). 

A multinodular growth pattern was identified in 20 pleomorphic adenomas (7.90%), while additional morphological changes included squamous metaplasia (37 cases; 14.62%), mild mitotic activity (24 cases; 9.48%), osseous metaplasia (five cases; 1.97%), ischemic necrosis (three cases; 1.18%), along with adipose metaplasia, cystic degeneration, and intravascular tumor emboli.

**Figure 5:**
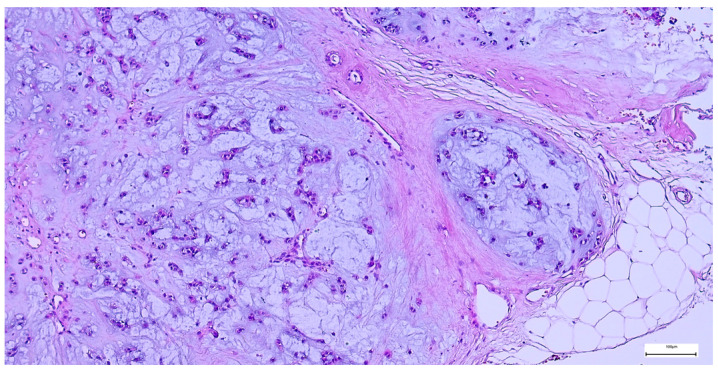
Satellite nodule of pleomorphic adenoma in close proximity to the main encapsulated tumor (H&E staining, 5×).

**Figure 6:**
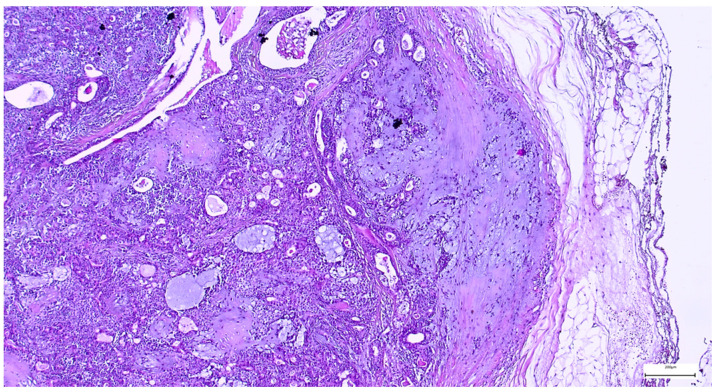
Pleomorphic adenoma with intact capsule and pseudopodial extensions protruding into the surrounding parotid parenchyma (H&E staining, 5×).

The capsule was incomplete in 67 cases (26.49%) of pleomorphic adenomas. Satellite nodules were documented as multiple nodules in 23 cases (9.09%), and as single nodules in 17 cases (6.71%). Pseudopodia were observed in 11 cases (4.34%), while both satellite nodules and pseudopodia were identified in 3 cases (1.27%) (Figure 5 and Figure 6).

**Figure 7:**
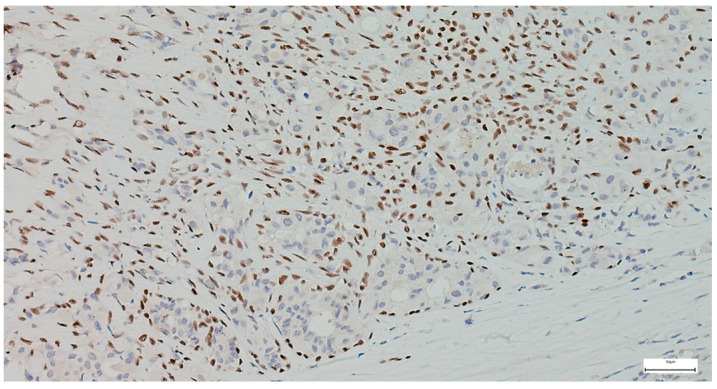
Myoepithelial cells show strong and diffuse nuclear p63 positivity in pleomorphic adenoma, with negative staining of luminal epithelial cells (IHC, 20×).

**Figure 8:**
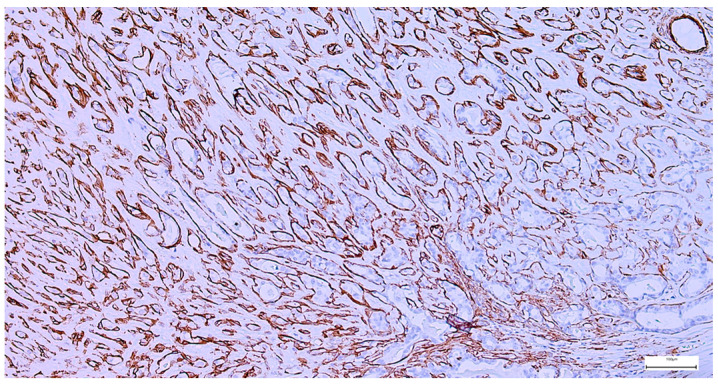
Peripheral myoepithelial cell immunohistochemical SMA positivity around cystic and tubular components in pleomorphic adenoma (IHC, 20×).

The immunohistochemical examination, performed for diagnostic certification in 29 cases of pleomorphic adenomas (11.46%), revealed CK7-positive immunoexpression of the luminal ductal epithelial cells, adding to p63 and S100 myoepithelial cells positivity. SMA, CD10, and CEA variable immunoexpression in myoepithelial and luminal epithelial cells was also registered (Figure 7 and Figure 8). AR and HER2 were negative in all the analyzed cases, while the Ki-67 proliferative index was low (<5%), confirming the benign type of proliferation.

**Figure 9:**
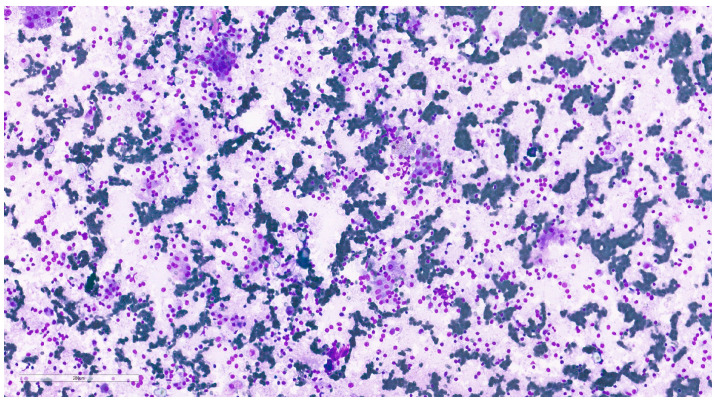
Pleomorphic adenoma cytological imprint showing numerous plasmacytoid myoepithelial cells with abundant, well-defined cytoplasm and ovoid nuclei with finely granular chromatin, dispersed in a fibrillary myxoid background (MGG, 20×).

**Figure 10:**
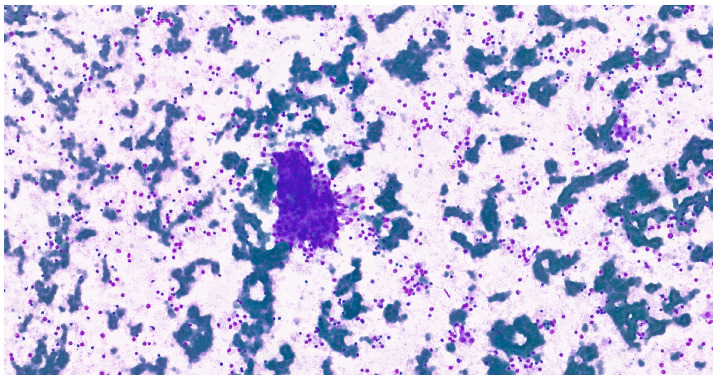
Cohesive epithelial clusters dispersed in a fibrillary myxoid background, consistent with the characteristic biphasic histological architecture in a cytological imprint of pleomorphic adenoma (MGG, 20×).

Intraoperative salivary imprint cytology demonstrated morphological features consistent with pleomorphic adenoma, in agreement with the final histopathological diagnosis in all cases (Figure 9 and Figure 10).

Tumor recurrence was rare in pleomorphic adenomas, being documented in four cases (1.58%) in a follow-up period ranging between 1 and 3 years, occurring predominantly in the parotid gland (1.18%), followed by the submandibular gland (0.39%). The gender distribution was uniform, with ages ranging between 15 and 50 years old. The tumors’ recurrences, detected in three patients, presented as numerous myxoid to fibrotic nodules of different sizes (22–45 mm diameter). 

**Figure 11:**
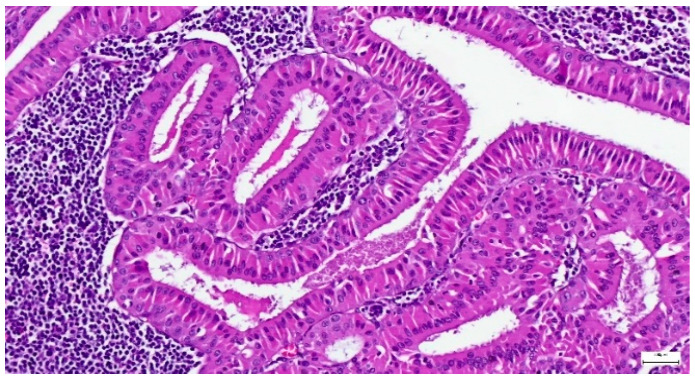
Warthin tumor showing a bilayered oncocytic epithelium with prominent papillary infoldings and a dense lymphoid stroma (H&E staining, 20×).

Among Warthin tumor histological subtypes, the infarcted/metaplastic variant was recorded in 18 cases (14.88%), while the common subtype has been identified in 103 cases (85.12%) (Table 3). Grossly, these appeared as well circumscribed masses, of 11–85 mm diameter, with solid areas, multiple cysts, and papillary projections on the cut section. The lesions exhibited a microscopic papillary-cystic proliferation, lined by a bilayered oncocytic epithelium, associated with a prominent lymphoid stroma (Figure 11). Additional morphologic changes included ischemic-type necrosis, along with squamous metaplasia (15 cases; 12.39%), and mucinous metaplasia (one case; 0.82%). Considering that their diagnosis was straightforward, an IHC investigation was not required.

All Warthin tumors had a unilateral location in our study group, exhibiting multifocal lesions in nine cases (7.43%), while the complete surgical excision was achieved in 108 cases (89.25%).

**Figure 12:**
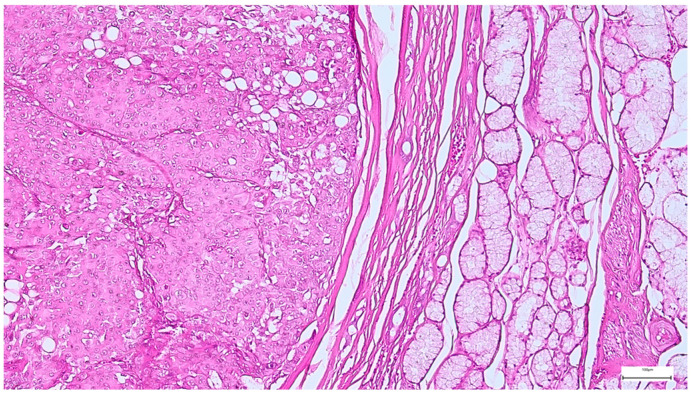
Well circumscribed, encapsulated myoepithelioma, with thick capsule, showing predominantly insular and solid architecture, along with focal trabecular areas (H&E staining, 10×).

Regarding myoepitheliomas, the major histological subtypes identified in our study group were epithelioid, documented in 10 cases (76.92%), and spindle cell subtype, registered in three cases (23.08%) (Figure 12). Most patients had SP (nine cases; 69.24%), and a complete capsule resection was registered in 12 cases (92.3%).

**Figure 13:**
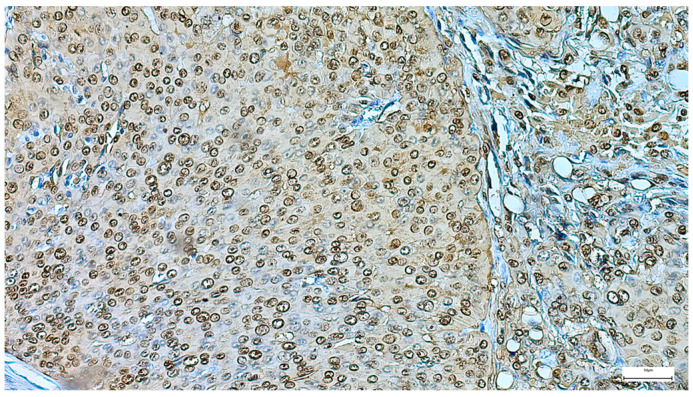
Myoepithelioma’s diffuse S100 positivity (IHC, 20×).

**Figure 14:**
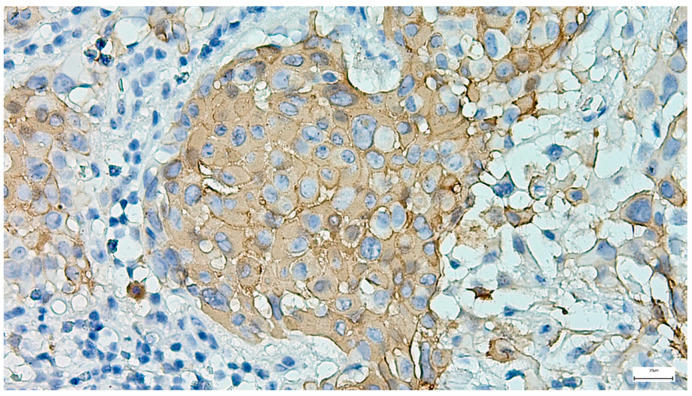
Diffuse cytoplasmic/membranous CK5/6 positivity in most cells of a myoepithelioma (IHC, 20×).

**Figure 15:**
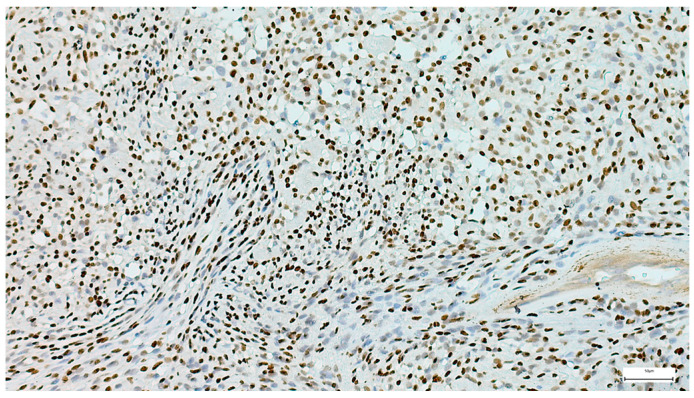
Strong and diffuse p63 nuclear positivity in a myoepithelioma (IHC, 20×).

A specific myoepithelial differentiation IHC panel was applied in selected cases for diagnosis confirmation, including S100, CK5/6, CD117, AE1/AE3, p63, calponin, and SMA (Figure 13, Figure 14 and Figure 15). Additional markers, such as ER, synaptophysin, and chromogranin, were negative. GFAP expression was observed in a subset of cases, supporting the myoepithelial nature of the tumor. The Ki-67 proliferation index was low in all cases, with values < 5%, consistent with the benign character of the lesions.

**Figure 16:**
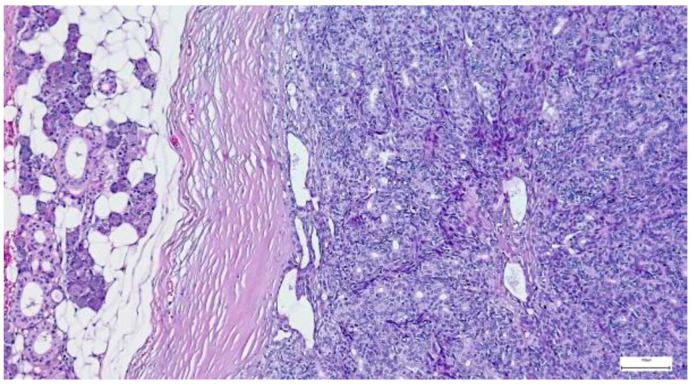
Basal cell adenoma composed of uniform basaloid cells arranged in a solid pattern, associated with a fibrocellular stroma (H&E staining, 10×).

Basal cell adenoma was identified in nine cases, all cases presenting as unilateral, rounded-ovoid, small-sized, encapsulated nodules, with a mean of 24.44 ± 9.82 mm. The lesions displayed variable histological patterns, along with cyst formation in three cases, the tumor cells being associated with a fibrocellular stroma (Figure 16). Complete excision was achieved in eight cases (88.89%), following SP in most cases (Table 3). IHC was performed in five cases (55.6%), highlighting the tumor cell population dual epithelial (AE1/AE3, CK7, and CD117 positive expression) and myoepithelial (p63, S100, and SMA positive expression) phenotype. AR was negative, and the Ki-67 proliferation index was ≤2% in all assessed cases.

Gross features of oncocytomas were those of well circumscribed, red to brown nodules, of 35.40 ± 8.50 mm mean size. The oncocytomas’ morphology showed an unspecified cellular subtype (three cases; 60.0%), added to clear cell variant in two cases (40.0%), in a fibrovascular stroma. A complete capsule resection was achieved in four tumors (80.0%), whereas a clear cell variant (20.0%) had an incomplete capsule. Surgical management consisted exclusively of SP, without any case requiring total parotidectomy or sialoadenectomy.

**Figure 17:**
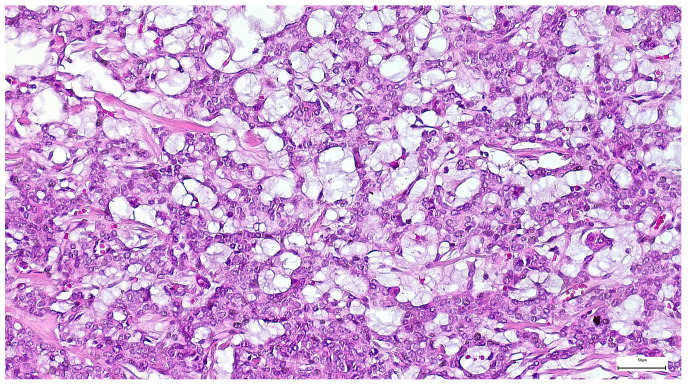
Tumor epithelial cells of a canalicular adenoma arranged in single and bilayered strands or anastomosing cords (H&E staining, 20×).

Only three cases of canalicular adenoma have been registered in our study, exhibiting well circumscribed single nodules, with a maximum diameter of 50 mm and complete capsular excision. The tumor epithelial cells were arranged in single or bilayered strands, anastomosing cords, canaliculi, or branching tubules, disposed in a loose connective stroma (Figure 17). The tumor showed CK7-positive immunoexpression, added to SMA-and p63-negative immunoexpression.

## 4. Discussion

Epidemiological data regarding salivary gland neoplasia show marked variability, reflecting significant geographical differences [9]. This variability may be attributed to the influence of environmental factors, added to different diagnostic approaches and the demographic characteristics of the population included in the research batches [5,10].

Previous studies have reported a higher susceptibility for the development of salivary gland tumors in women, although the gender differences may be variable according to the tumor histological type [5,10,11,12,13,14]. In contrast, other relatively recent studies have shown either a balanced gender distribution or a slight male predominance [2,15]. Our study revealed a slight predominance of female patients diagnosed with benign tumors, corresponding to a women-to-men ratio of 1.12:1, supporting most of the previous studies [14,16].

Analysis of the age group distribution currently shows that the incidence of benign tumors progressively increases with aging, with a peak observed in the fifth and sixth decades of life [14,17], although previous findings demonstrated that most benign salivary gland tumors occurred between the fourth and sixth decades of life, representing approximately 38–54% of cases, with patients’ diagnostic ages ranging from 45 to 52 years old [14,16,17] and a mean diagnostic age of 49.1 years [18]. The slightly higher rate of patients registered in the sixth and seventh decades of life in our study supports recent reports regarding the frequent development of benign salivary gland tumors in older people.

Multiple studies have shown that benign salivary gland tumors follow a relatively consistent anatomical distribution, with the parotid glands being most commonly involved [4,19], with an incidence range between 52.8% and 83.7% [6,13], followed by the submandibular glands, representing 10–15% and, less frequently, the minor salivary glands. Furthermore, minor salivary gland tumors, whether benign or malignant, are relatively rare, comprising approximately 10–15% of all salivary gland tumors, mostly occurring in the palate and lips [20]. Nevertheless, a retrospective analysis found that only 2.5% of benign salivary gland tumors arise in this location [21]. In line with these findings, a recent study of 105 salivary gland lesions in a Romanian population reported that only 4.62% of benign neoplasms involved the minor salivary glands [22], data which are consistent with our results.

Benign epithelial tumors account for approximately 80% of salivary gland neoplasms, encompassing a wide variety of histological types [16,23], exhibiting a slow growth and a favorable prognosis, although there is a risk of recurrence or malignant transformation [24,25]. The underlying mechanisms of malignant transformation are regulated by a variety of different factors, such as oncogenes, cell-cycle regulators, angiogenesis factors, viral infections, and cytokines [7,26]. The findings of different population-based studies have reported a predominance of benign tumors within the salivary gland tumors, with rates between 64.72% [5] and 89% [10]. Benign epithelial tumors accounted for 71% of all epithelial salivary gland tumors in our study group, in agreement with data reported in the literature.

The most frequent types of benign salivary gland tumors are pleomorphic adenomas and Warthin tumors, with a variable distribution in different studies, with relatively consistent rates regardless of geographic or demographic characteristics. In this respect, large retrospective analyses have reported that pleomorphic adenomas account for approximately 65.6% of cases, while Warthin tumors represent about 29.2%, based on cohorts exceeding 2400 cases [27,28,29]. However, multi-center studies have reported that pleomorphic adenomas represent approximately 70% of benign salivary gland tumors, while Warthin tumors account for about 17%, suggesting some degree of regional variation [4]. In addition, other population-based analyses have found pleomorphic adenomas to comprise up to 70.5% of benign tumors, reinforcing the dominant distribution of this histological pattern across different cohorts [30]. While pleomorphic adenoma is well-recognized as the most prevalent benign tumor, some reports have identified other tumors, such as basal cell adenoma (2% to 7%) or myoepithelioma (1% to 3%), as the second most frequent benign histologic types in specific populations [3,31,32,33,34]. This general distribution is in agreement with our findings, demonstrating a predominance of pleomorphic adenoma and Warthin tumor histological types, with a cumulated rate of 92.57%, followed by myoepithelioma (3.21%), basal cell adenoma (2.22%), oncocytoma (1.23%), and canalicular adenoma (0.74%). Although this distribution complies with data from the literature, some population-specific patterns have been noticed, highlighting common diagnostic challenges reported in recent studies.

Numerous epidemiological studies have reported a higher prevalence (60–70%) of pleomorphic adenoma in female patients, with female-to-male ratios ranging from 1.38:1 to 2:1, suggesting a possible gender-related susceptibility of this tumor subtype [18,19,31,32]. By contrast, Warthin tumor exhibits a strong male predominance, being often associated with smoking [10], representing 80–90% of cases, yielding to a male-to-female ratio of 1.5:1 [35]. Moreover, it is well-established that patients with obesity and metabolic syndrome show a higher risk of cardiovascular and inflammatory diseases or neoplasms development, including Warthin tumor [26,36,37]. A characteristic feature of obese patients is the disruption of adipokine and pro-inflammatory cytokine secretion by expanded adipose areas, leading to systemic inflammation that may affect various organs, including salivary glands [37]. In this context, individuals with Warthin tumors have a statistically significant higher body mass index (BMI) compared to those with other benign parotid gland tumors [38]. Additionally, an increase in salivary pro-inflammatory factors in obese patients, such as interleukin 15 (IL-15), pentraxin 3 (PTX-3), monocyte chemoattractant protein 1 (MCP-1), and tumor necrosis factor-α receptors 1 and 2 (TNF-α-R1, TNF-α-R2) has been detected, suggesting that the Warthin tumor may have an inflammatory etiology [39]. However, consistent prospective studies with multivariate analysis are necessary to explore the impact of the pro-inflammatory cascade in relation to increased BMI to validate obesity as a significant factor in Warthin tumor development. 

Regarding other tumor types, myoepithelioma showed no significant gender difference, while oncocytoma exhibited a slight female predominance, representing 54–57% of cases [3,29]. Although our findings revealed a similar gender-related distribution, confirming an evident female predominance of pleomorphic adenoma (39.85% in women vs. 22.77% in men) and a strong male predominance of Warthin tumor (20.29% in men vs. 9.65% in women), both myoepithelioma and oncocytoma showed a slight male propensity in our cohort, probably related to our study limitation or to population-based characteristics.

Concerning the patients’ age distribution, pleomorphic adenoma is predominantly diagnosed in adults aged 30–60 years, with a peak incidence in the fourth and fifth decades of life and a mean age-group between 43 and 48 years [18,31]. In total, 41.83% of pleomorphic adenomas in our study group were diagnosed, in descending order of prevalence, within the 50–59, 60–69, and 40–49 age groups, with a peak diagnosis in the sixth decade of life, not in the fifth decade of life as reported in the literature, corresponding to specific population characteristics. Conversely, Warthin tumor is most commonly diagnosed in older adults, with a mean age group of 60–70 years, with more than 80% of cases older than 55 years [10]. The distribution was similar in our study group, with 11.38% of Warthin tumors identified in patients in the 60–69 age group.

The majority of studies regarding pleomorphic adenomas identified their location in the parotid gland (80–85%), with less tumors developed in the submandibular gland (8–10%) and minor salivary glands (5–7%) [8,18,31], while Warthin tumors almost exclusively occur in the parotid gland (>95%), with bilateral tumors in 5–10% and multiple tumors in up to 20% of cases [10]. Pleomorphic adenomas were predominantly located in the parotid gland (86.16%) in our cohort, followed by the submandibular and minor salivary glands, while most Warthin tumors (95.04%) were confined to the parotid gland, confirming their well-recognized topographical distribution. Furthermore, the strong tissue-specific affinity of pleomorphic adenoma and Warthin tumor for the parotid gland may suggest a potential biological tropism [35], also supported by our study results, with 82.42% of registered cases falling into these two tumor types.

Myoepitheliomas are diagnosed in a broader age range, with a peak between 50 and 60 years, with rare occurrences in patients younger than 30 years [3,28]. A similar distribution was observed in our study group, with a higher frequency in middle-aged and older patients. Similarly, oncocytomas are almost exclusively diagnosed in older adults, with a mean age of 58.7 years, and over 75% of cases are diagnosed in individuals older than 50 years [29]. This type of tumor was a rare histological finding in our study group, being diagnosed in only five cases, exhibiting an exclusive occurrence in the 50–79-year-old age range. Other tumor types, such as basal cell adenoma and canalicular adenoma, are rare benign salivary epithelial tumors [40,41], in agreement with our findings, with only 2.97% of cases registered in this category. Taken together, these results comply with the age-specific characteristics reported in the literature and support the notion that the aging process is significant in the clinical onset of benign salivary gland tumors.

Although rare, myoepitheliomas occur predominantly in the parotid gland (40–50% and 66%, respectively), followed by minor salivary glands, notably the palate and buccal mucosa [3,32]. Myoepitheliomas were predominantly located in the parotid gland, with rare lesions identified in minor salivary glands, in our cohort, consistent with their anatomical distribution patterns. Their location in minor salivary glands led to the hypothesis that the local glandular microenvironment may influence their development [42], a hypothesis that may also be related to our study results. Similarly, oncocytomas show a strong parotid gland propensity (66%), with a reduced rate arising in the submandibular gland (23.3%) and in the minor salivary glands (26.6%) [43]. The exclusive parotid gland location in our study group may be related to the limitations of the study, to the population characteristics, or to the involvement of other microenvironment influences compared to myoepithelioma tumorigenesis.

Canalicular adenoma accounts for less than 1% of all salivary gland tumors [44], with approximately 80% of reported cases in the upper lip and extremely uncommon in the major salivary glands [41]. In this regard, a retrospective analysis of 430 cases identified the upper lip and the hard palate as the most common sites of canalicular adenoma occurrence (66.3% and 14.5%, respectively) [44]. However, canalicular adenoma and basal cell adenomas were exclusively identified in the parotid gland in our series (0.74% of cases). Therefore, considering that the parotid gland canalicular adenoma has a low incidence, with only a few reported cases [45], our findings are significant for the knowledge related to this rare entity.

Statistical analysis revealed a significant association in our study group between histological subtype and location (*p* = 0.004), as well as the association with patient age and gender, supporting the hypothesis that demographic and anatomical factors collectively influence the morphological phenotype and the clinical presentation of benign salivary gland tumors. 

Regarding the characteristic histopathological features, pleomorphic adenoma contains biphasic epithelial–myoepithelial components in a chondromyxoid stroma, frequently showing focal or exuberant squamous metaplasia, with keratin-filled cysts mimicking mucoepidermoid carcinoma [46,47]. Mild mitotic activity can occur in otherwise typical pleomorphic adenoma, whereas brisk or atypical mitoses are considered worrisome features and prompt careful exclusion of carcinoma ex-pleomorphic adenoma [48]. Moreover, osseous metaplasia [49] and cystic degeneration are uncommon, leading to differential diagnostic challenges with cystic salivary neoplasms [50]. Moreover, intravascular tumor plugs or emboli have been rarely identified in pleomorphic adenoma, which represents a diagnostic pitfall, considering that vascular involvement is reported in the setting of metastasizing pleomorphic adenoma [51,52]. Squamous metaplasia was frequently identified in our series, while mild mitotic activity was detected in a minority of cases. Moreover, IHC performed in selected cases (focused on ductal–myoepithelial panel, AR negativity, and low Ki-67 index) allowed for the differential diagnosis with adenoid cystic, epithelial–myoepithelial, or squamous cell carcinomas.

Although the microscopic diagnosis is usually facile in cases of Warthin tumor, challenges may arise if areas of ischemic coagulative necrosis, mucinous metaplasia, and squamous metaplasia of the oncocytic epithelium are noticed, leading to a differential diagnosis with malignancies [53,54]. Additionally, a significant diagnostic pitfall is the ‘Warthin-like’ variant of mucoepidermoid carcinoma (WL-MEC), diagnosed by p63- and CK5/6-positive expression, added to the presence of MAML2 gene rearrangement [55]. Squamous or mucinous metaplasia were rarely identified in our cohort (12.39% and 0.82%, respectively), supporting the diagnosis of Warthin tumor in the absence of further ancillary investigations.

Although representing only 1–2% of all salivary gland tumors, oncocytoma warrants a careful differential diagnosis, mainly of its clear cell phenotype with other clear cell salivary tumors or metastases [56,57,58]. The oncocytoma’s IHC profile is useful for differential diagnosis, typically showing CK7, EMA, and anti-mitochondrial antibody (AMA) positivity, along with a low proliferative index [59,60,61]. NR4A3 negativity is useful in differential diagnosis with acinic cell carcinoma, and GATA3 positivity differentiates it from salivary duct carcinoma [62]. However, the two cases of clear cell variant oncocytomas registered in our study did not raise differential diagnosis issues.

In the fifth WHO framework, oncocytic carcinoma is no longer retained as an independent salivary entity. However, carcinomas may frequently display a diffuse oncocytic phenotype, being classified by their characteristic features (e.g., AR-positive salivary duct carcinoma, MEC with MAML2 rearrangements, secretory carcinoma with ETV6 NTRK3 gene fusion, and DOG1- and SOX10-positive acinic cell carcinoma). Additionally, recent reviews provide practical oncocytoid tumors diagnostic algorithms, emphasizing key differences from nodular oncocytic hyperplasia, presenting as an unencapsulated, multifocal lesion [8,56,62]. Positioning our investigated cases within this framework allowed for their inclusion in the benign category and sets up the key exclusion criteria, requiring ancillary testing.

Another histological type, salivary gland myoepithelioma, representing a pure myoepithelial proliferation, raises differential diagnosis challenges with myoepithelial carcinoma, basal cell adenoma, plasmacytoma, or pleomorphic adenoma. However, the myoepithelial markers (S100, SOX10, p63, calponin, and SMA) added to CD117, synaptophysin, and chromogranin negativity, and Ki-67 low-index are valuable tools for diagnosis, including in our study group.

Along with myoepithelioma, basal cell adenoma and canalicular adenoma are rare salivary tumors. Basal cell adenoma occurs most frequently in the parotid gland, followed by the minor salivary glands and the nasal septum, typically not exceeding 30 mm in size [63]. These findings are consistent with our results, with only 2.22% cases represented by parotid gland basal cell adenoma, with a mean diameter of 24.44 ± 9.82 mm. The ductal cells markers (AE1-/AE3- and CK7-positive expression) and basaloid cells markers (p63-, S100-, and SMA-positive expression), added to a low Ki-67 proliferation index (≤2%), supported its differential diagnosis with adenoid cystic carcinoma, basal cell carcinoma, or canalicular adenoma. 

Regarding the tumor histogenesis, benign epithelial salivary gland tumors arise from ductal, acinar, or myoepithelial epithelium within the major or minor salivary glands, rather than representing mesenchymal or hematolymphoid primary or secondary involvement [53,64]. The primary versus secondary differential has direct clinical relevance considering the numerous intraparotid and periparotid lymph nodes that receive afferent lymphatic drainage from the scalp, face, and external ear [65]. The terminology also prevents confusion with salivary gland type neoplasms that arise in other organs, e.g., lung and breast, but only mimic salivary histology [66,67]. Consistent with this site of origin, the same benign epithelial histotypes may present as primary tumors if salivary-type seromucinous glands or ectopic salivary tissue are present. In addition to the major and minor salivary glands, primary pleomorphic adenoma may arise in the external auditory canal, from ceruminous glands, without any continuity with the salivary glands [68]. Other locations may be the tra-cheobronchial tree and lungs, from the submucosal seromucinous glands [69,70], or the sinonasal tract, including the nasal cavity, the nasal septum, and the maxillary sinus [71,72,73,74,75], and in the posterior fossa. All these rare locations may be attributed to heterotopic salivary tissue [76]. Similarly, a Warthin tumor may present as a primary larynx lesion [77,78], while basal cell adenoma may present as a primary lesion of the nasal cavity or nasal septum [79,80]. According to literature guides [68,69,70,77,78,79], we considered a lesion as primary if no anatomical continuity with a major salivary gland was noticed, if native salivary type glands were present, and if the histology features matched a benign salivary epithelial phenotype.

The intraoperative imprint cytology has been validated as a rapid adjuvant technique that achieves a high diagnostic concordance with the final histopathology diagnosis [81]. The large series of cases demonstrates that intraoperative consultation reliably distinguishes benign from malignant disease in salivary gland surgery, with high specificity and overall accuracy [81]. However, recent reviews emphasize common pitfalls (e.g., extensive squamous or mucinous metaplasia, cystic change, or oncocytic pattern) that may blur benign–malignant boundaries and require correlation with the overall matrix and cell population [82]. Although performed in selective cases in our study, the intraoperative salivary imprint cytology represented an adjuvant diagnostic method that allowed the differentiation of pleomorphic adenoma from other salivary gland tumors and provided real-time accuracy for surgical decision-making. 

Although epithelial benign salivary gland tumors have a favorable course following the standard surgical resection, local tumor recurrences, with a recurrence rate below 2–3% or malignant transformation, are sometimes registered, especially in pleomorphic adenoma [24,25]. Pleomorphic adenoma recurrences usually occur within 18 months of the initial parotidectomy and in relatively young patients [24,83], a finding consistent with our study results. Although the complex mechanism of recurrences is elusive, surgical technique deficiencies, along with some tumor histopathological features, may have an important contribution. In this context, different studies reported that myxoid stroma, tumor rupture, incomplete capsule excision, satellite nodules, and pseudopodia are the most significant pathological features associated with an increased risk of pleomorphic adenoma recurrence [24,84,85,86,87,88]. Demonstrating the validity of these histopathological risk factors, pseudopodia have been identified in 55% of tumors of a pleomorphic adenoma series with subsequent recurrence and in only 8% of patients without recurrences [24,89]. Additionally, satellite nodules have been observed in 60% of patients with recurrent pleomorphic adenomas compared with 10% in non-recurrent cases [84]. Our findings comply with these data, with satellite nodules being identified in 50% and pseudopodia in 25% of recurrent pleomorphic adenomas in our study group.

The incomplete capsule excision and large tumor size may also facilitate the pleomorphic adenoma recurrence. In this context, large tumors (>40 mm) display incomplete capsules more often, a frequent myxoid stroma, and satellite nodules, features that significantly increase the tumor recurrence risk [24]. These data are concordant with our observation, with pleomorphic adenoma recurrences registered in two cases exhibiting tumors exceeding 40 mm in diameter.

Recurrence may also be registered in Warthin tumors, usually due to inadequate tumor surgical excision or in a multifocal lesion [35,88], with a single report of a tumor recurrence (0.84%), after 120 months of follow-up, in a recent study [90]. Additionally, apparent “recurrences” after limited surgery often represent second primaries in a multifocal gland rather than true regrowth [35,91,92,93]. Although multifocal lesions were registered in 7.43% of cases, no Warthin tumor recurrence was registered in our study, in agreement with data from the literature. Furthermore, our 89.25% margin-negative rate complies with published series showing durable local control after complete excision of benign parotid tumors and supports the current shift toward limited resections, e.g., ECD or SP.

Salivary gland oncocytoma displays a low risk for local recurrence, most probably related to incomplete tumor excision [43,56,57]. In this context, a recent systematic review reported a pooled recurrence of about 2.7%, at approximately 34.7 months [43]. The management of these tumors exclusively consisted of SP in our study group, with no recurrences documented during the follow-up, with durable local control, including in a clear-cell case with incomplete encapsulation.

Considering that salivary myoepitheliomas are well circumscribed and frequently encapsulated, although the capsule may sometimes be absent, a complete excision may be easily achieved. Although small benign peripheral protrusions and satellite nodules can be noted, they should not be over-interpreted as malignancy [94,95]. In our cohort, a complete encapsulation was observed in 92.30% of cases, supporting the benign architecture and providing a durable local control when complete excision was achieved. However, considering the potential recurrence of myoepithelioma and canalicular and basal cell adenoma, a long-term follow-up is essential.

Although improved WHO guidelines [8] may lead to a reclassification of salivary gland tumors, supported by immunohistochemistry and molecular tests, this may be applied mainly in malignancies, considering that these new criteria did not lead to diagnostic changes in the analyzed cohort of benign tumors in our study.

Additionally, differences between cytological and final histological diagnoses may be related to limited sampling and overlapping morphological features, highlighting the importance of a comprehensive diagnostic approach [30], supported by our study results.

The limitations of our study related to the data collection from a single center are overcome by the large patient addressability from our region to this facility and the extensive period of time of data collection. However, our study advises the implementation of regional data collection for salivary gland tumors in the National Cancer Registry in Romania and alignment with European standards.

Taken together, our study adds valuable data supporting the predictive value of demographical, anatomical, and histopathological factors in the diagnosis of salivary gland lesions and highlights the necessity to consider regional variability in epidemiological data interpretation, which is in agreement with other studies [8,31]. Moreover, due to limited published data [22,96] and an extensive period of research, our study provides a general perspective on the epidemiological and pathological profile of epithelial benign salivary gland tumors in our geographical area.

## 5. Conclusions

This retrospective study provides a relevant contribution to the understanding of benign salivary gland tumor characteristics and behavior in Romania. Our findings confirm their higher frequency compared to malignant lesions and comply with the previous demographic and epidemiological studies, regarding the dominant pleomorphic adenoma and Warthin tumor histological types diagnosis, most of them with a parotid gland location. Although the study was performed at a tertiary center, the long period of time for case collection and their follow-up over a fifteen-year period allowed for the identification of extremely rare histological types, i.e., canalicular adenoma, or of the characteristics of relatively rare recurrences of benign salivary gland tumors.

A slight female susceptibility was generally noted in our cohort, while the age groups seemed significantly correlated with tumor development, with the highest frequency in older ages than those reported in literature, in the sixth and seventh decades of life, adding important data to the characteristics of benign salivary gland tumors in the population of north-eastern Romania.

Furthermore, other differences between the tumors’ clinicopathological findings in our study and data from the literature may also be related to specific population-based characteristics. Additionally, key features, such as capsular irregularity with occasional extracapsular pseudopodia in pleomorphic adenoma, an oncocytic cystic architecture in Warthin tumor, and compact basaloid patterns in basal cell adenoma, follow age- and gland-specific distributions. These morphology-based observations support a better preoperative stratification, more confident margin assessment, and an individualized extent of excision, with the goal of gland function preservation, thus supporting a conservative surgical approach in these patients.

The distinctive morphologic features of each histological type are not only useful for young pathologists-in-training but also, supplemented by ancillary testing in selected cases, may lead to an accurate classification of benign salivary gland tumors and to a personalized follow-up of patients with recurrence risk factors.

## Figures and Tables

**Table 1:** Epithelial salivary gland benign tumors distribution by gender and location in the study group.

	Buccal Glands (*n*; %)	Palatine Glands (*n*; %)	Right Parotid Gland (*n*; %)	Left Parotid Gland (*n*; %)	Sublingual Gland (*n*; %)	Right Submandibular Gland (*n*; %)	Left Submandibular Gland (*n*; %)	Total (*n*; %)
**Female**	1 (0.24%)	5 (1.23%)	106 (26.23%)	83 (20.54%)	1 (0.24%)	10 (2.47%)	8 (1.98%)	214 (52.97%)
**Male**	-	5 (1.23%)	78 (19.30%)	92 (22.77%)	-	6 (1.48%)	9 (2.22%)	190 (47.03%)
**Total**	1 (0.24%)	10 (2.47%)	184 (45.54%)	175 (43.31%)	1 (0.24%)	16 (3.96%)	17 (4.20%)	404 (100%)

*n*—number of cases.

**Table 2:** Distribution of benign epithelial salivary gland tumors by histological type, location, and patient’s age group.

			Pleomorphic Adenoma	Warthin Tumor	Myoepithelioma	Basal Cell Adenoma	Oncocytoma	Canalicular Adenoma	*p* Value *
			(*n*; %)	(*n*; %)	(*n*; %)	(*n*; %)	(*n*; %)	(*n*; %)	
**Gender**	F		161 (39.85%)	39 (9.65%)	5 (1.23%)	6 (1.48%)	2 (0.51%)	1 (0.24%)	<0.001
	M		92 (22.77)	82 (20.29%)	8 (1.98%)	3 (0.74%)	3 (0.76%)	2 (0.49%)	
**Age group**	10–19		4 (0.99%)	-	-	-	-	-	<0.001
	20–29		21 (5.19%)	-	-	-	-	-	
	30–39		38 (9.40%)	1 (0.24%)	-	-	-	-	
	40–49		47 (11.63%)	12 (2.97%)	2 (0.51%)	-	-	-	
	50–59		72 (17.82%)	36 (8.91%)	-	2 (0.51%)	1 (0.25%)	-	
	60–69		50 (12.37%)	46 (11.38%)	8 (1.98%)	6 (1.48%)	1 (0.25%)	1 (0.24%)	
	70–79		20 (4.95%)	22 (5.44%)	3 (0.74%)	1 (0.24%)	3 (0.76%)	1 (0.24%)	
	80–89		1 (0.24%)	4 (0.99%)	-		-	1 (0.24%)	
**Location**	Parotid glands	L	104 (25.74%)	58 (14.35%)	5 (1.23%)	4 (0.99%)	4 (0.99%)	1 (0.24%)	0.004
		R	114 (28.21%)	57 (14.10%)	4 (0.99%)	5 (1.23%)	1 (0.24%)	2 (0.51%)	
	Submandibular glands	L	12 (2.97%)	5 (1.23%)	-	-	-	-	
		R	14 (3.46%)	1 (0.24%)	1 (0.24%)	-	-	-	
	Sublingual glands		-	-	1 (0.24%)	-	-	-	
	Palatine glands		8 (1.98%)	-	2 (0.51%)	-	-	-	
	Buccal salivary glands		1 (0.24%)	-	-	-	-	-	
	Total		253 (62.62%)	121 (29.95%)	13 (3.21%)	9 (2.22%)	5 (1.23%)	3 (0.74%)	

*n*—number of cases; F—female; M—male; L—left; R—right. * Chi-square test: *p* < 0.05 is significant.

**Table 3:** Pathological features and surgical management of benign epithelial salivary gland tumors in our study group.

	Pleomorphic Adenoma		Warthin Tumor		Myoepithelioma		Basal Cell Adenoma		Oncocytoma		**Canalicular** **Adenoma**	
	(*n*; %)		(*n*; %)		(*n*; %)		(*n*; %)		(*n*; %)		**(*n*; %)**	
**Variant** **(Subtype)**	Common	248 (98.02%)	Common	103 (85.12%)	Epithelioid	10 (76.92%)	Tubulo-trabecular	4 (44.44%)	Common	3 (60%)	Common	3 (100%)
	Oncocytic	5 (1.98%)	Infarcted/ Metaplastic	18 (14.88%)	Fusiform	3 (23.08%)	Solid	5 (55.56%)	Clear cell	2 (40%)		
**Tumor size (mm)**	Mean SD (min; max)	32.55 13.38 (9; 82)		31.75 13.70 (11; 85)	-	25.92 7.29 (12; 38)	-	24.44 9.82 (10; 37)	-	35.40 8.50 (26; 45)	-	28.67 18.71 (15; 50)
**Stroma**	Myxoid	138 (54.54%)	Lymphoid	121 (100%)	Collagenous	13 (100 %)	Fibrocellular	9 (100%)	Fibro-vascular	5 (100%)	Loose	3 (100%)
	Chondro-myxoid	72 (28.45%)										
	Chondroid	17 (6.71%)										
	Others	26 (10.28%)										
**Other** **pathological** **features**	Single satellite nodule	17 (6.71%)	Squamous metaplasia	15 (12.39%)	-	-	Cyst formation	3 (33.33%)	-	-	-	-
	Multiple satellite nodules	23 (9.09%)	Mucinous metaplasia	1 (0.82%)								
	Pseudo- podia	11 (4.34%)										
**Capsule**	CCE	186 (73.51%)	CCE	108 (89.25%)	CCE	12 (92.30%)	CCE	8 (88.89%)	CCE	4 (80%)	CCE	3 (100%)
	PM	67 (26.49%)	PM	13 (10.75%)	PM	1 (7.70%)	PM	1 (11.11%)	PM	1 (20%)	PM	-
**Surgery resection** **type**	ECD	38 (15.02%)	ECD	29 (23.97%)	ECD	-	ECD		ECD	-	ECD	-
	SP	142 (56.12%)	SP	86 (71.07%)	SP	9 (69.24%)	SP	7 (77.78%)	SP	5 (100%)	SP	3 (100%)
	TP	38 (15.02%)	TP	-	TP	-	TP	2 (22.22%)	TP	-	TP	-
	TS	26 (10.28%)	TS	6 (4.95%)	TS	2 (15.38%)	TS	-	TS		TS	-
	WLE	9 (3.55%)	WLE	-	WLE	2 (15.38%)	WLE	-	WLE	-	WLE	-
**Tumor** **recurrence**	No	249 (98.42%)	No	121 (100%)	No	13 (100%)	No	9 (100%)	No	5 (100%)	No	3 (100%)
	Yes	4 (1.58%)	Yes	-	Yes	-	Yes	-	Yes	-	Yes	-
**Total**	253 (62.62%)		121 (29.95%)		13 (3.21%)		9 (22.27%)		5 (1.23%)		3 (0.74%)	

CCE—complete capsule excision; ECD—extracapsular dissection; n—number of cases; max—maximum; min—minimum; PM—positive margin; SD—standard deviation; SP—superficial parotidectomy; TP—total parotidectomy; TS—total sialoadenectomy; WLE—wide local excision.

## Data Availability

The original contributions presented in this study are included in the article. Further inquiries can be directed to the corresponding authors.

## References

[1] Gontarz M., Bargiel J., Gąsiorowski K., Marecik T., Szczurowski P., Zapała J., Wyszyńska-Pawelec G. (2021). Epidemiology of Primary Epithelial Salivary Gland Tumors in Southern Poland—A 26-Year, Clinicopathologic, Retrospective Analysis. J. Clin. Med..

[2] Tian Z., Li L., Wang L., Hu Y., Li J. (2010). Salivary gland neoplasms in oral and maxillofacial regions: A 23-year retrospective study of 6982 cases in an eastern Chinese population. Int. J. Oral Maxillofac. Surg..

[3] da Silva L.P., Serpa M.S., Viveiros S.K., Sena D.A.C., Pinho R.F.d.C., Guimarães L.D.d.A., Andrade E.S.d.S., Pereira J.R.D., da Silveira M.M.F., Sobral A.P.V. (2018). Salivary gland tumors in a Brazilian population: A 20-year retrospective and multicentric study of 2292 cases. J. Cranio-Maxillofac. Surg..

[4] Alsanie I., Rajab S., Cottom H., Adegun O., Agarwal R., Jay A., Graham L., James J., Barrett A.W., van Heerden W. (2022). Distribution and Frequency of Salivary Gland Tumours: An International Multicenter Study. Head Neck Pathol..

[5] Gao M., Hao Y., Huang M.X., Ma D.Q., Chen Y., Luo H.Y., Gao Y., Cao Z.Q., Peng X., Yu G.Y. (2017). Salivary gland tumours in a northern Chinese population: A 50-year retrospective study of 7190 cases. Int. J. Oral Maxillofac. Surg..

[6] Ghaderi H., Kruger E., Ahmadvand S., Mohammadi Y., Khademi B., Ghaderi A. (2023). Epidemiological Profile of Salivary Gland Tumors in Southern Iranian Population: A Retrospective Study of 405 Cases. J. Cancer Epidemiol..

[7] Costin C.A., Chifu M.B., Pricope D.L., Grigoraş A., Balan R.A., Amălinei C. (2024). Are HPV oncogenic viruses involved in salivary glands tumorigenesis?. Rom. J. Morphol. Embryol..

[8] WHO Classification of Tumours Editorial Board (2024). Head and Neck Tumours.

[9] McKenzie J., Lockyer J., Singh T., Nguyen E. (2023). Salivary gland tumours: An epidemiological review of non-neoplastic and neo-plastic pathology. Br. J. Oral Maxillofac. Surg..

[10] Aegisdottir A.L., Tryggvason G., Jonsdottir A.M., Jonasson J.G. (2020). Salivary gland tumours in Iceland 1986–2015: A nationwide epidemiological analysis over a 30-year time period. APMIS.

[11] Mejia-Velazquez C., Duran-Padilla M., Gomez-Apo E., Quezada-Rivera D., Gaitan-Cepeda L. (2012). Tumors of the salivary gland in mexicans. A retrospective study of 360 cases. Med. Oral Patol. Oral Cir. Bucal..

[12] Torabinia N., Khalesi S. (2014). Clinicopathological study of 229 cases of salivary gland tumors in Isfahan population. Dent. Res. J..

[13] Cunha J.L., Coimbra A.C., Silva J.V., Nascimento I.S., Andrade M.E., Oliveira C.R., Almeida O.P., Soares C.D., Sousa S.F., Albuquerque-Júnior R.L. (2020). Epidemiologic analysis of salivary gland tumors over a 10-years period diagnosed in a northeast Brazilian population. Med. Oral Patol. Oral Cir. Bucal.

[14] Cunha J.L.S., Hernandez-Guerrero J.C., de Almeida O.P., Soares C.D., Mosqueda-Taylor A. (2020). Salivary Gland Tumors: A Retrospective Study of 164 Cases from a Single Private Practice Service in Mexico and Literature Review. Head Neck Pathol..

[15] Subhashraj K. (2008). Salivary gland tumors: A single institution experience in India. Br. J. Oral Maxillofac. Surg..

[16] de Oliveira F.A., Duarte E.C., Taveira C.T., Maximo A.A., de Aquino E.C., Alencar Rde C., Vencio E.F. (2009). Salivary gland tu-mor: A review of 599 cases in a Brazilian population. Head Neck Pathol..

[17] Zanwar P.R., Humbe J.G., Mandale M.S., A Nandkhedkar V., Wagh S.P. (2024). Clinico-epidemiological profile of salivary gland tumours: An institutional study. IP J. Diagn. Pathol. Oncol..

[18] Ansari M.H. (2007). Salivary Gland Tumors in an Iranian Population: A Retrospective Study of 130 Cases. J. Oral Maxillofac. Surg..

[19] Ungureanu L.-B., Ghiciuc C.-M., Costan V.V., Ungureanu C., Ianole V., Apostol D.-G.C. (2025). Benign and Malignant Parotid Gland Tumors: Insights from a Five-Year Northeast Romanian Population. J. Clin. Med..

[20] Vicente O.P., Marqués N.A., Aytés L.B., Escoda C.G. (2008). Minor salivary gland tumors: A clinicopathological study of 18 cases. Med. Oral Patol. Oral Cir. Bucal..

[21] Banerjee R., Chandrakar R.K. (2020). Histomorphological Study of Salivary Gland Tumours. Int. J. Curr. Res. Rev..

[22] Vamesu S., Ursica O.A., Gurita A.M., Voda R.I., Deacu M., Aschie M., Bosoteanu M., Cozaru G.C., Mitroi A.F., Orasanu C.I. (2023). A retrospective study of nonneoplastic and neoplastic disorders of the salivary glands. Medicine.

[23] Israel Y., Rachmiel A., Ziv G., Nagler R. (2016). Benign and malignant salivary gland tumors - clinical and demographic characteris-tics. Anticancer Res..

[24] Dulguerov P., Todic J., Pusztaszeri M., Alotaibi N.H. (2017). Why Do Parotid Pleomorphic Adenomas Recur? A Systematic Review of Pathological and Surgical Variables. Front. Surg..

[25] Valstar M.H., Mast H., Hove I.T., Moonen L.R., Balm A.J., E Smeele L., Koljenović S., Dinjens W.N., van Velthuysen M.F. (2021). Malignant transformation of salivary gland pleomorphic adenoma: Proof of principle. J. Pathol. Clin. Res..

[26] Grigoraș A., Amalinei C. (2023). Multi-Faceted Role of Cancer-Associated Adipocytes in Colorectal Cancer. Biomedicines.

[27] Eveson J.W., Cawson R.A. (1985). Tumours of the minor (oropharyngeal) salivary glands: A demographic study of 336 cases. J. Oral Pathol. Med..

[28] Ansari M., Askarpour S., Nasrollahi H., Mohammadianpanah M., Ahmadloo N., Omidvari S., Mosalaei A., Hamedi S.H., Zare-Bandamiri M. (2022). Clinical and Pathological Features and Outcome of Patients with Salivary Gland Cancer a Single Centre Report. Asian Pac. J. Cancer Care.

[29] Sando Z., Fokouo J.V., Mebada A.O., Djomou F., NDjolo, Oyono J.L. (2016). Epidemiological and histopathological patterns of salivary gland tumors in Cameroon. Pan Afr. Med. J..

[30] Irani S., Dehghan A., Kalvandi Z. (2023). Correlation of Clinical and Histopathological Features of Salivary Pleomorphic Adenoma. J. Dent. (Shiraz).

[31] Taghavi N., Sargolzaei S., Mashhadiabbas F., Akbarzadeh A., Kardouni P. (2015). Salivary gland tumors: A 15- year report from iran. Turk. Patoloji. Derg..

[32] Saghravanian N., Ghazi N., Saba M. (2013). Clinicopathologic evaluation of salivary gland neoplasms: A 38-year retrospective study in Iran. Ann. Diagn. Pathol..

[33] Bradley P.J., McGurk M. (2013). Incidence of salivary gland neoplasms in a defined UK population. Br. J. Oral Maxillofac. Surg..

[34] Ichihara T., Kawata R., Higashino M., Terada T., Haginomori S.-I. (2014). A more appropriate clinical classification of benign parotid tumors: Investigation of 425 cases. Acta Oto-Laryngologica.

[35] Quer M., Hernandez-Prera J.C., Silver C.E., Casasayas M., Simo R., Poorten V.V., Guntinas-Lichius O., Bradley P.J., Tong-Ng W., Rodrigo J.P. (2021). Current Trends and Controversies in the Management of Warthin Tumor of the Parotid Gland. Diagnostics.

[36] Grigoras A., Amalinei C. (2025). The Role of Perirenal Adipose Tissue in Carcinogenesis—From Molecular Mechanism to Therapeu-tic Perspectives. Cancers.

[37] Gontarz M., Bargiel J., Gąsiorowski K., Marecik T., Szczurowski P., Hramyka A., Kuczera J., Wieczorkiewicz A., Wyszyńska-Pawelec G. (2024). Could Obesity Be Related to the Increasing Incidence of Warthin Tumors?. J. Clin. Med..

[38] Kadletz L., Grasl S., Perisanidis C., Grasl M.C., Erovic B.M. (2019). Rising incidences of Warthin’s tumors may be linked to obesity: A single-institutional experience. Eur. Arch. Otorhinolaryngol..

[39] Lehmann A.P., Nijakowski K., Swora-Cwynar E., Łuczak J., Czepulis N., Surdacka A. (2020). Characteristics of salivary inflamma-tion in obesity. Pol. Arch. Intern. Med..

[40] Kanaujia S.K., Singh A., Nautiyal S., Ashutosh K. (2015). Basal Cell Adenoma of Parotid Gland: Case Report and Review of Literature. Indian J. Otolaryngol. Head Neck Surg..

[41] Thompson L.D., Bauer J.L., Chiosea S., McHugh J.B., Seethala R.R., Miettinen M., Muller S. (2015). Canalicular adenoma: A clini-copathologic and immunohistochemical analysis of 67 cases with a review of the literature. Head Neck Pathol..

[42] Motallebnejad M., Seyedmajidi M., Babaoli O.K., Yarmand F. (2015). Oncocytoma of palatal minor salivary gland. Arch. Iran. Med..

[43] Alberto P.L., Ashim S., Megan K., Wei Z., Nestor G., Matthew Z., Dayana M., Marcelo V., John W. (2024). Salivary gland onco-cytomas. A systematic review. Head Neck Pathol..

[44] Peraza A.J., Wright J., Gómez R. (2017). Canalicular adenoma: A systematic review. J. Cranio-Maxillofac. Surg..

[45] Azar A., Alkheder A., Salam R., Elnasser M.S., Alahmad V., Hajjar F. (2024). Canalicular Adenoma in the Parotid Gland: A Rare Case Study. Ear Nose Throat J..

[46] Hosur M.B., Puranik R.S., Dandagi S.G., Patil V.M. (2024). Pleomorphic adenoma with extensive squamous metaplasia and keratinizing cysts: Diagnostic and clinical pitfalls—A report of two cases and review of literature. J. Oral Maxillofac. Pathol..

[47] Gaskin D.A., Reid A., Gaskin P.S. (2022). Pleomorphic adenoma with extensive squamous metaplasia: The first well-documented case involving the submandibular gland. Hum. Pathol. Rep..

[48] de Lima-Souza R.A., Bělohlávková K., Michal M., Altemani A., Mariano F.V., Skálová A. (2025). Atypical and worrisome histological features in pleomorphic adenoma: Challenging and potentially significant diagnostic pitfall. Virchows Arch..

[49] Gubod E.R., Ramanathan A., Chong Mei Yee S., Tilakaratne W.M. (2022). Bone Formation in Pleomorphic Adenoma: A Case Re-port. Cureus.

[50] Khetrapal S., Jetley S., Hassan M.J., Jairajpuri Z. (2015). Cystic Change in Pleomorphic Adenoma: A Rare Finding and a Diagnostic Dilemma. J. Clin. Diagn. Res..

[51] Skalova A., Altemani A., Di Palma S., Simpson R.H., Hosticka L., Andrle P., Laco J., Toner M., Vozmitsel M.A., Szakacs S. (2012). Pleomorphic adenoma of the salivary glands with intravascular tumor deposits: A diagnostic pitfall. Am. J. Surg. Pathol..

[52] Watson M., McAllister P., Conn B., MacNeill M., Handley T.P.B. (2018). Metastasising Pleomorphic Salivary Adenoma: A Rare Case Report of a Massive Untreated Minor Salivary Gland Pleomorphic Adenoma with Concurrent Ipsilateral Cervical Node Metastases. Head Neck Pathol..

[53] Skálová A., Hyrcza M.D., Leivo I. (2022). Update from the 5th Edition of the World Health Organization Classification of Head and Neck Tumors: Salivary Glands. Head Neck Pathol..

[54] Aoki R., Tanaka T. (2024). Pathogenesis of Warthin’s Tumor: Neoplastic or Non-Neoplastic?. Cancers.

[55] Bieńkowski M., Kunc M., Iliszko M., Kuźniacka A., Studniarek M., Biernat W. (2020). MAML2 rearrangement as a useful diagnostic marker discriminating between Warthin tumour and Warthin-like mucoepidermoid carcinoma. Virchows Arch..

[56] Skalova A., Bradova M., Da Cruz Paula A., Faquin W.C. (2025). Oncocytic Tumors in the Salivary Gland: A Tri-Focal Review - Integrated Cytopathological, Pathological, and Molecular Features. Acta Cytol..

[57] Parmar R., Kalaria A.N., A Patel K. (2024). Oncocytic Lesions of Salivary Glands: Morphological, Immunohistochemical, and Molecular Findings. Cureus.

[58] Zhu W., Zhang Y., Li F., Li G., Zhang P., Fang H., Bian L. (2024). Case of clear-cell oncocytoma of parotid gland and literature review. Hua Xi Kou Qiang Yi Xue Za Zhi.

[59] Ozolek J.A., Bastacky S.I., Myers E.N., Hunt J.L. (2005). Immunophenotypic comparison of salivary gland oncocytoma and meta-static renal cell carcinoma. Laryngoscope.

[60] de Paiva J.P.G., Kirschnick L.B., Roldán D.G., Martins M.D., Santos-Silva A.R., Soares C.D., Jorge J. (2025). Clinicopathological Study of Oncocytomas of Head and Neck Region: A Systematic Review. J. Oral Pathol. Med..

[61] Faur A.C., Buzaș R., Lăzărescu A.E., Ghenciu L.A. (2024). Current Developments in Diagnosis of Salivary Gland Tumors: From Structure to Artificial Intelligence. Life.

[62] Katabi N. (2024). Oncocytoid Salivary Tumors: Differential Diagnosis and Utility of Newly Described Immunohistochemistry. Head Neck Pathol..

[63] Al Qooz F., Alanazi M., Al Olaimat M.S., Malahmeh T., Alzoubi Z.R. (2024). Submandibular basal cell adenoma – A rare presenta-tion. Human Pathol. Rep..

[64] van Herpen C., Poorten V.V., Skalova A., Terhaard C., Maroldi R., van Engen A., Baujat B., Locati L., Jensen A., Smeele L. (2022). Salivary gland cancer: ESMO–European Reference Network on Rare Adult Solid Cancers (EURACAN) Clinical Practice Guideline for diagnosis, treatment and follow-up. ESMO Open.

[65] Pecorari G., Pizzo C., Briguglio M., Cravero E., Riva G. (2023). Primary and Secondary Tumors of the Parotid Gland: Clinical Features and Prognosis. Cancers.

[66] Takamori S., Yatabe Y., Osoegawa A., Aokage K., Yoshioka H., Miyoshi T., Mimae T., Endo M., Hattori A., Yotsukura M. (2023). Rare but clinically important salivary gland-type tumor of the lung: A review. Ultrasound Med. Biol..

[67] Schwartz C.J., Krings G. (2024). Salivary gland-like tumors of the breast: Histopathologic and genetic features with clinical implications. Semin. Diagn. Pathol..

[68] Saito C., Kanazawa T., Yamaguchi T., Nakamura K.-I., Ichimura K. (2014). Primary Pleomorphic Adenoma of the External Auditory Canal: A Case Report and Review of the Literature. Case Rep. Otolaryngol..

[69] Požgain Z., Dulić G., Kristek J., Rajc J., Bogović S., Rimac M., Kiš I. (2016). Giant primary pleomorphic adenoma of the lung presenting as a post-traumatic pulmonary hematoma: A case report. J. Cardiothorac. Surg..

[70] Fitchett J., Luckraz H., Gibbs A., O’KEefe P. (2008). A Rare Case of Primary Pleomorphic Adenoma in Main Bronchus. Ann. Thorac. Surg..

[71] Basharat R., Bjorling A., Samara G. (2024). Pleomorphic Adenoma of the Nasal Cavity. Cureus.

[72] Konsulov S., Milkov D., Markov D., Poryazova E.G. (2024). Diagnostic Challenges of Sinonasal Pleomorphic Adenoma. Cureus.

[73] Pan X., Cheng X., Wang Q., Feng W., Lu Y., Wei X. (2024). Pleomorphic Adenoma in the Nasal Cavity: Case Report and Review of Literature. Ear Nose Throat J..

[74] Alshammasi R., Jones H., Walsh M., Kruseman N., McDermott M., Robinson I., Colreavy M. (2024). Pediatric sinonasal pleo-morphic adenoma: A case report. J. Rhinol..

[75] Chęciński M., Nowak Z. (2022). Maxillary Sinus Pleomorphic Adenoma: A Systematic Review. Surgeries.

[76] Yang L., Tan L., Lau Q., Jayalath R. (2016). Rare Primary Pleomorphic Adenoma in Posterior Fossa. World Neurosurg..

[77] Tascan I.S., Yenigun A., Ozkan F., Dogan M. (2023). Locational and Clinical Varieties of Warthin Tumor: Two Rare Case Presentations. Ear Nose Throat J..

[78] Nisa L., Landis B.N., Salmina C., Ailianou A., Karamitopoulou E., Giger R. (2015). Warthin’s tumor of the larynx: A very rare case and systematic review of the literature. J. Otolaryngol.-Head Neck Surg..

[79] Doo J.G., Lee H., Jeon S.Y., Kim J.S. (2023). Basal Cell Adenoma With Atypia in the Nasal Cavity Mimicking a Malignant Tumor. Ear Nose Throat J..

[80] Wang Q., Chen H., Wang S. (2015). Basal cell adenoma of nasal septum: Report of a case and review of literature. Int. J. Clin. Exp. Pathol..

[81] Jaiswal Y., Gadkari R. (2020). Evaluation of role of intraoperative cytology technique in diagnosis and management of cancer. J. Cytol..

[82] Saoud C., Bailey G.E., Graham A., Bonilla L.M., Sanchez S.I., Maleki Z. (2024). Pitfalls in Salivary Gland Cytology. Acta Cytol..

[83] Manucha V., Ioffe O.B. (2008). Metastasizing Pleomorphic Adenoma of the Salivary Gland. Arch. Pathol. Lab. Med..

[84] Park G.C., Cho K., Kang J., Roh J., Choi S., Kim S.Y., Nam S.Y. (2012). Relationship between histopathology of pleomorphic adenoma in the parotid gland and recurrence after superficial parotidectomy. J. Surg. Oncol..

[85] Mantsopoulos K., Iro H. (2021). Tumour spillage of the pleomorphic adenoma of the parotid gland: A proposal for intraoperative measures. Oral Oncol..

[86] Iro A.-K., Agaimy A., Müller S.K., Sievert M., Iro H., Mantsopoulos K. (2021). Satellite nodules in pleomorphic adenomas of the parotid gland: A nightmare for less invasive parotid surgery?. Oral Oncol..

[87] Pagnani G., Palma A., Bozza F., Lombardi C.M.R., Becelli R. (2025). Systematic Review and Case Report on the Surgical Management of Pleomorphic Adenomas: Lessons on Recurrence and Error Prevention. J. Clin. Med..

[88] Zanghì A., Cavallaro A., Marchi M., La Via L., Sanfilippo F., Cappellani A., Di Majo S., Zanghì A. (2025). Surgical management of benign tumors of the parotid gland: The advantages of extracapsular dissection compared to traditional surgical techniques. Front. Surg..

[89] Henriksson G., Westrin K.M., Carlsoo B., Silfverswärd C. (1998). Recurrent primary pleomorphic adenomas of salivary gland origin. Cancer.

[90] Lee D.H., Yoon T.M., Lee J.K., Lim S.C. (2019). Surgical treatment strategy in Warthin tumor of the parotid gland. Braz. J. Otorhinolaryngol..

[91] Rimmer R.A., Cottrill E.E. (2018). Multifocal Warthin’s Tumor: An Uncommon Presentation of Bilateral Cervical Lymphadenopathy. Case Rep. Otolaryngol..

[92] Orabona G.D., Bonavolontà P., Iaconetta G., Forte R., Califano L. (2013). Surgical Management of Benign Tumors of the Parotid Gland: Extracapsular Dissection Versus Superficial Parotidectomy—Our Experience in 232 Cases. J. Oral Maxillofac. Surg..

[93] Maiorano E., Muzio L.L., Favia G., Piattelli A. (2002). Warthin’s tumour: A study of 78 cases with emphasis on bilaterality, multifocality and association with other malignancies. Oral Oncol..

[94] Telugu R.B., Gaikwad P., Baitule A., Michael R.C., Mani T., Thomas M. (2020). Myoepithelial Tumors of Salivary Gland: A Clinicopathologic and Immunohistochemical Study of 15 Patients with MIB-1 Correlation. Head Neck Pathol..

[95] Thompson L.D.R., Xu B. (2023). Top Ten Differentials to Mull Over for Head and Neck Myoepithelial Neoplasms. Head Neck Pathol..

[96] Gidea-Paraschivescu E., Luca R.E., Ratiu C.A., Roi C.I. (2025). Parotid Gland Tumors: An Institutional 8-Year Retrospective Study Spanning the COVID-19 Pandemic and Global Diagnostic Trends. J. Clin. Med..

